# Habituation of the startle reflex depends on attention in cannabis users

**DOI:** 10.1186/s40359-016-0158-8

**Published:** 2016-10-26

**Authors:** Karina K. Kedzior, Eileen Wehmann, Mathew Martin-Iverson

**Affiliations:** 1 Institute of Psychology and Transfer, University of Bremen, (FB 11), Grazer Str. 2c, 28359 Bremen, Germany; 2 School of Engineering and Science, Jacobs University Bremen, Bremen, Germany; 3 Clinical Neurophysiology Unit, Graylands Hospital and Pharmacology & Anaesthesiology Unit, School of Medicine & Pharmacology, Faculty of Medicine, Dentistry & Health Sciences, The University of Western Australia, Perth, Australia

**Keywords:** Startle habituation, Cannabis misuse, Selective attention

## Abstract

**Background:**

Cannabis use is associated with an attention-dependent deficit in prepulse inhibition of the startle reflex (PPI). The aim of the current study was to investigate startle habituation in cannabis users and healthy controls during two attentional tasks.

**Methods:**

Auditory startle reflex was recorded from orbicularis oculi muscle while participants (12 controls and 16 regular cannabis users) were either attending to or ignoring 100 dB startling pulses. Startle habituation was measured as the absolute reduction in startle magnitude on block 2 (last nine trials) vs. block 1 (first nine trials).

**Results:**

Startle habituation with moderate effect sizes was observed in controls and cannabis users only while they were ignoring the startling pulses but not while they were attending to them. Similar results were also observed in controls (lifetime non-users of cannabis) and cannabis users with lifetime cannabis use disorders (CUD).

**Conclusion:**

Startle habituation appears to depend on selective attention but not on cannabis use. Startle habituation was present when attention was directed away from auditory startling pulses in healthy controls and cannabis users. Such a similar pattern of results in both groups suggests that at least a trend exists towards presence of startle habituation regardless of cannabis use or CUD in otherwise healthy members of the general population.

## Background

The relationship between cannabis use and mental health has been studied extensively. Empirical data show that particularly heavy cannabis use is positively related to affective outcomes, including major depression [1] and anxiety disorders [2]. While the magnitude of these relationships remains small for affective outcomes [3], there exists a more consistent and stable association between cannabis use and psychotic outcomes [4]. It has been shown that early onset and heavy cannabis use is related to earlier onset and higher odds for psychosis and is especially prevalent in younger, male, first-episode patients with schizophrenia [5–8].

Regardless of such extensive research, the physiological bases of the relationship between cannabis use and psychotic outcomes remain largely unknown. One candidate for studying such physiological bases is the process of sensorimotor gating which is thought to indirectly measure the allocation of cognitive resources to appropriately filter the sensory stimuli [9]. Sensorimotor gating can be quantified as prepulse inhibition (PPI) of the startle reflex [10]. Startle reflex is a contraction of the skeletal and facial muscles in response to a sudden, relatively intense stimulus (startling pulse) in any sensory modality [11]. PPI is a reduction in startle magnitude which occurs when a low-intensity stimulus (prepulse) is presented 30–500 ms before the startling pulse [12]. Apart from prepulses, startle magnitude can be modified by selective attention [13]. Furthermore, in the absence of prepulses, startle magnitude habituates (is reduced) over time after repetitive presentation of startling pulses [14].

Sensorimotor gating appears to be affected by psychosis and cannabis use. In general, schizophrenia studies have shown that, relative to healthy controls, PPI deficit was observed either during passive (no task) paradigms [15] or during selective attention paradigms depending on attentional demand ([13, 16], for review see [17]). In addition, participants with cannabis-induced psychotic disorder showed PPI deficits but only at very short prepulse-pulse intervals relative to healthy controls [18]. PPI was also reduced in participants at high risk for psychosis with urinary cannabinoids relative to healthy controls [9]. In contrast, studies of cannabis users without psychosis reported less consistent PPI deficits. During passive attention paradigms adult cannabis users and non-user controls showed similar levels of PPI [19], while adolescent cannabis users failed to maintain PPI over time compared to controls [20]. The evidence from studies with selective attention paradigms suggests that PPI deficit occurred only while cannabis users attended to, but not ignored, auditory pulses relative to controls [21, 22]. Interestingly, studies directly comparing cannabis users and schizophrenia patients showed that PPI deficits were similar in both groups relative to controls. Specifically, PPI deficit was reported in both cannabis users and in (non-user) schizophrenia patients while attending to, but not ignoring, pulses and prepulses during various attentional tasks [23, 24].

Unlike PPI, another aspect of startle modification, namely startle habituation, received less research attention, particularly in cannabis users. Habituation refers to a reduction in behavioral response following repeated stimulation and does not involve sensory or motor fatigue [25]. Startle habituation is often quantified as a reduction in startle magnitude on blocks of trials towards the end compared to the beginning of the experiment. In schizophrenia research startle habituation has been used to explore information processing and attentional deficits associated with this disorder [26]. Unlike PPI deficits, only some schizophrenia studies reported a deficit (or a trend towards a deficit) in startle habituation during passive attention paradigms (for example, [14, 27–35]) while others did not find such a deficit (for example, [36–38]). Such inconsistent results are not surprising given the heterogeneous methods of quantifying startle habituation [39]. It is also unclear if and how startle habituation is altered by cannabis use. The evidence from passive attention paradigms showed that both controls and cannabis users (healthy or at high risk for psychosis) displayed similar patterns of startle habituation in terms of reduction in startle magnitude on later relative to earlier trials [9, 19, 20]. To our knowledge startle habituation has not been studied during selective attention paradigms in cannabis users, although similarly to PPI, startle habituation might depend on attention. Attentional processing is particularly affected in heavier and longer-term cannabis users [40]. Thus, if startle habituation depends on attention, it might be especially affected by heavier cannabis use. Although neither PPI nor startle habituation can be used as physiological markers of psychosis or cannabis use, it is important to study these indirect measures of brain function to develop effective therapies against psychiatric disorders [10] and to understand the physiological bases of the relationship between cannabis use and psychosis. The aim of the current study was to investigate startle habituation in cannabis users relative to healthy controls during two selective attention tasks involving either attending to or ignoring auditory pulses. The second aim was to investigate startle habituation in heavier cannabis users (users with lifetime cannabis use disorders, CUD) relative to healthy controls. It was hypothesized that, similarly to attention-related PPI deficits, startle habituation might be impaired in cannabis users relative to controls but only while attending to pulses and not when ignoring them [21]. It was also expected that, if cannabis use affects attention [40], any deficit in startle habituation would be particularly evident in heavier cannabis users with lifetime CUD relative to healthy controls.

## Methods

The current methods have already been described in detail elsewhere [21, 23]. The data reported in this study have not been published before. The study was approved by the research ethics committees at the University of Western Australia and Graylands Hospital, Perth, Australia, and all participants gave a written informed consent to take part in the study.

### Participants

Participant recruitment procedure, exclusion criteria, and demographic characteristics of both groups are shown elsewhere [21, 23]. Briefly, following the exclusion of participants positive for other substances in urine and/or with symptoms of psychiatric disorders the sample consisted of 12 healthy controls and 16 cannabis users recruited from the general population of Perth, Australia. All controls were non-users of cannabis in the last 12 months. The majority of cannabis users (81 %; 13/16) were daily-weekly users in the last 12 months, 69 % (11/16) reported lifetime symptoms of CUD, and 75 % (12/16) reported recent (24 h) use and were positive for cannabinoids in urine [21].

### Cannabis use and CUD diagnoses

Cannabis use was defined as at least one-time use of cannabis (in any form, concentration, or duration) in the last 12 months since the testing session. Self-reports regarding the recent use of cannabis (within 24 h) were validated with urine screens and were found to be accurate in the current participants [41]. Lifetime diagnoses of CUD (cannabis dependence and/or abuse) were established based on DSM-IV and/or ICD-10 criteria using the Composite International Diagnostic Interview (CIDI-Auto 2.1) [42]. The presence of CUD diagnoses on CIDI-Auto 2.1 was accurately predicted using scores on the lifetime Severity of (Cannabis) Dependence Scale, SDS [43], in the current participants [44]. Although it cannot be ruled out, it was assumed that our participants had little motivation to misreport their substance use based on the high agreements among self-reports of recent use and urine screens, among lifetime CUD diagnoses and SDS scores, as well as the full anonymity and strict confidentiality of the study [41, 44]. Since withdrawal from other substances (such as caffeine) can affect startle habituation [45], all participants were required to maintain their usual cannabis consumption (if users) and to refrain from nicotine for at least 1 h before testing and alcohol on the day of testing.

### Startle procedure

The auditory startle reflex was measured during two attentional tasks. The current study focuses on 36 pulse-alone trials only (18 per attentional task). During the Attend Task the participants were asked to passively listen to the background white noise (60 dB) interrupted by 18 pulses at 100 dB (white noise; duration 50 ms, nearly instantaneous rise/fall time) presented binaurally via headphones. During the Ignore Task the participants were told to ignore the auditory stimuli and play a handheld Tetris-like computer game. The order of attentional tasks (Attend – Ignore or Ignore – Attend) was counterbalanced within each group.

### Data acquisition and processing

A detailed description of data acquisition and processing can be found elsewhere [21]. The startle reflex was acquired as electromyogram (EMG) from the left orbicularis oculi muscle. The magnitude of the startle reflex was measured as the area under the peak curve (μV) to take into account both the magnitude and the duration of startle response.

### Data analysis

The mean startle magnitudes were computed for each participant on the first half (block 1 with nine trials) and the second half of the experiment (block 2 with nine trials) using IBM-SPSS 22.0. Startle habituation was measured as the absolute difference in the mean startle magnitude between block 1 and block 2 on each attentional task and in each group. Group means were compared using the repeated measures analysis of covariance (ANCOVA) with two within-subject factors (ATTENTION with two levels: Attend vs. Ignore Tasks; BLOCK with two levels: 1 vs. 2), one between-subject factor (GROUP with two levels: controls vs. cannabis users), and two covariates (cigarettes per day and alcoholic drinks per week in the last 12 months). Covariates were used because, relative to controls, cannabis users reported significantly higher nicotine and alcohol consumption in the last 12 months [21]. ANCOVA was followed up with pairwise comparisons corrected for family-wise error using Bonferroni’s adjustment. The effect sizes for pairwise comparisons were computed using the standardized mean difference, Hedges’ *g*, for paired or independent means [46]. The interpretation criteria for the absolute size of Hedges’ *g* are: .20–.49 (small effect), .50–.79 (moderate effect), and ≥ .80 (large effect) [46].

## Results

### Participant characteristics: controls vs. cannabis users

The two groups (controls and cannabis users) were matched on demographic characteristics (gender, handedness, age, IQ, education) and caffeine use [21] while, relative to controls, cannabis users reported significantly higher nicotine and alcohol consumption in the last 12 months [21].

### Startle habituation: controls vs. cannabis users

According to aim 1 of the current study, startle habituation was investigated in cannabis users relative to controls during two attentional tasks. The results of ANCOVA are shown in Table 1.

**Table 1:** Startle habituation in controls vs. cannabis users

	*MS*	*df*	*F*	*p*_*two-tailed*_	Power
Between subject-effect					
GROUP (G)	44686.22	1	.04	.844	.05
Error	1136543.52	24			
Within subject-effects					
BLOCK (B)	1867751.11	1	10.07	.004*	
B × G	14118.78	1	.08	.785	.06
Error	185411.16	24			
ATTENTION (A)	11425152.19	1	22.88	<.001*	
A × G	1432.61	1	.003	.958	.05
Error	499442.31	24			
Interactions					
B × A	12044.68	1	.12	.737	.06
B × A × G	3554.59	1	.03	.855	.05
Error	104706.22	24			

Note. Effect sizes are reported in text and on figures

*Abbreviations*: *df* degrees of freedom, *MS* mean square

*p < .05

#### Group

**Fig. 1:**
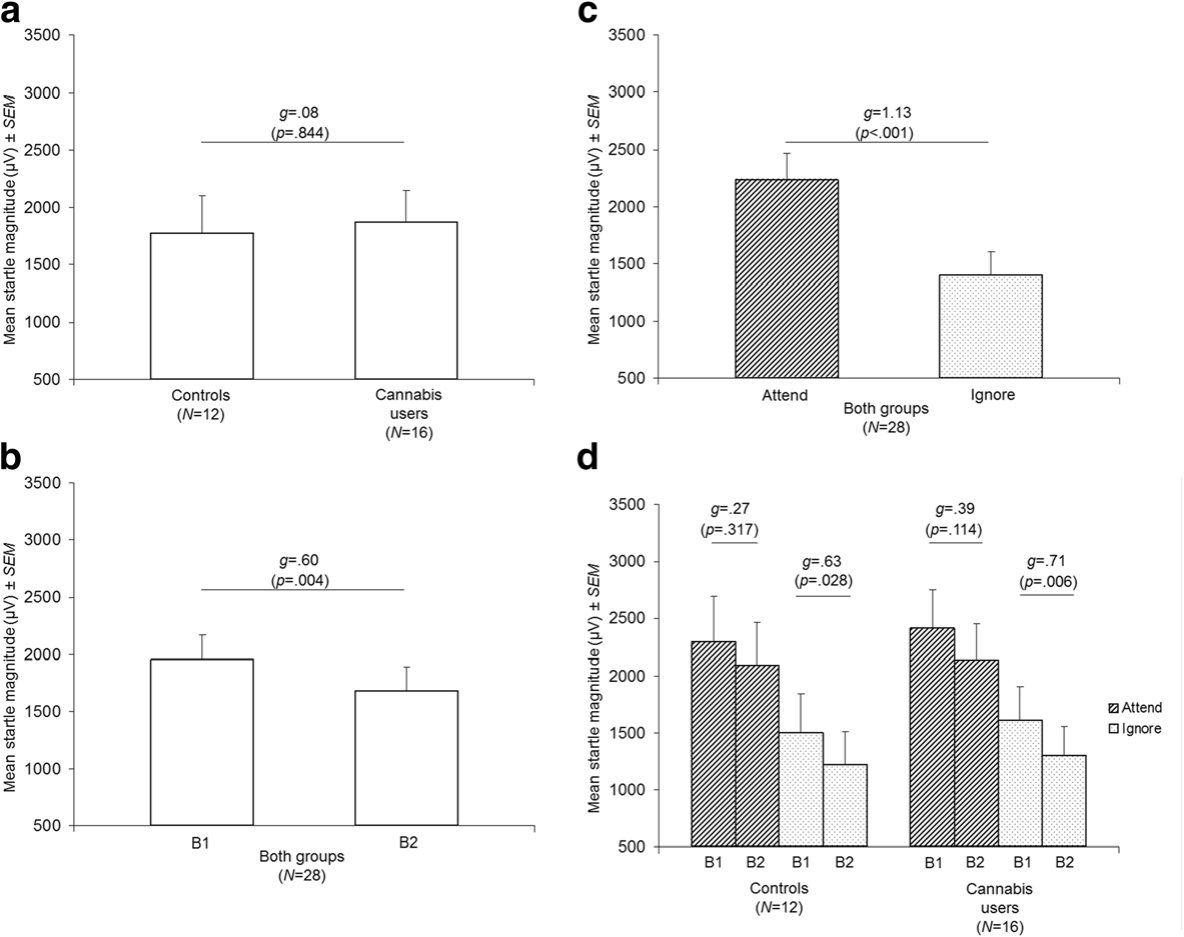
Mean startle magnitudes adjusted for nicotine and alcohol use depending on **a** group (controls vs. cannabis users), **b** block (1 vs. 2), **c** attention (Attend vs. Ignore Tasks), and **d** group, block, and attention. All *p*-values were adjusted using Bonferroni’s correction. Abbreviations: B1, Block 1; B2, Block 2; *g*, standardized mean difference (Hedges’ *g*; effect size); *N*, sample size; *SEM*, standard error of the mean

The main effect of GROUP was not statistically significant (Table 1). The difference in mean startle magnitudes adjusted for nicotine and alcohol use was negligible between controls and cannabis users (*g* = .08) (Fig. 1a).

#### Habituation

There was a statistically significant main effect of BLOCK (Table 1). The inspection of means adjusted for nicotine and alcohol use revealed that startle habituation occurred because startle magnitudes were significantly reduced on block 2 relative to block 1 (Fig. 1b). The effect size of startle habituation was only moderate (*g* = .60; Fig. 1b).

#### Attentional modulation

There was a statistically significant main effect of ATTENTION (Table 1). The inspection of means adjusted for nicotine and alcohol use revealed that startle magnitudes were significantly reduced on the Ignore Task relative to the Attend Task (Fig. 1c). The effect size of attentional modulation of startle magnitudes was large (*g* = 1.13; Fig. 1c).

#### Habituation by group and attention

A similar pattern of startle habituation was observed in controls and cannabis users. Pairwise comparisons revealed that startle habituation depends on attention in both groups (Fig. 1d). Startle habituation with moderate effect sizes was observed only on the Ignore Task in controls (*g* = .63) and in cannabis users (*g* = .71; Fig. 1d). In contrast, startle habituation on the Attend Task had only small effect sizes (*g* = .27–.39) in both groups (Fig. 1d).

### Participant characteristics: controls (lifetime non-users of cannabis) vs. cannabis users with CUD

Demographic characteristics of controls (lifetime non-users of cannabis) and cannabis users with lifetime CUD) are shown in Table 2. Both groups were matched on all characteristics except for nicotine and alcohol consumption in the last 12 months which were significantly higher in cannabis users relative to controls (Table 2).

**Table 2:** Participant characteristics depending on lifetime CUD (dependence/abuse)

Demographics	Controls no CUD	Cannabis users with CUD	*χ* ^*2*^ *(df)*	*p*_*two-tailed*_
*N*	7	11		
Male/Female	6/1	8/3	.42 (1)	.518
Right/Left-handed	5/2	9/2	.27 (1)	.605
	*M ± SD* (range)	*M ± SD* (range)	*t (df)*	*p*_*two-tailed*_
Age (years)	34 ± 9 (18–43)	29 ± 8 (19–44)	1.23 (16)	.238
IQ	104 ± 10 (86–115)	103 ± 10 (79–117)	.25 (16)	.809
Education (years)	13 ± 2 (10–14)	13 ± 2 (9–17)	-.64 (16)	.531
Alcohol/week (last 12 months)	3 ± 3 (0–10)	10 ± 8 (0–24)	−2.53 (15)	.023*
Cigarettes/day (last 12 months)	0 ± 0 (0–.1)	3 ± 1 (0–10)	−2.42 (10)	.036*
Coffee cup/day (last 12 months)	4 ± 1 (0–15)	2 ± 1 (0–10)	.56 (16)	.586
Duration of use (years)	-	13 ± 8 (2–27)		
Age of first use (years)	-	16 ± 3 (13–21)		
Last use (hours before testing)	-	9 ± 5 (2–13)		
Urine cannabinoids (μg/l)	-	692 ± 910 (0–2000)		
SDS score	-	4 ± 2 (0–7)		

Note*.* All controls were lifetime non-users of cannabis

*Abbreviations*: *CUD* lifetime cannabis use disorder, *df* degrees of freedom, *SDS* Severity of (Cannabis) Dependence Scale (15 indicates maximum dependence on cannabis)

*p < .05

### Startle habituation: controls vs. cannabis users with CUD

According to aim 2 of the current study, startle habituation was investigated in cannabis users with lifetime CUD relative to controls (lifetime non-users of cannabis) during two attentional tasks. The results of ANCOVA are shown in Table 3.

**Table 3:** Startle habituation in controls vs. cannabis users with lifetime CUD

	*MS*	*df*	*F*	*p*_*two-tailed*_	Power
Between subject-effect					
GROUP (G)	90864.23	1	.10	.759	.06
Error	929419.40	14			
Within subject-effects					
BLOCK (B)	1176010.10	1	4.65	.049*	
B × G	49900.05	1	.20	.664	.07
Error	253131.49	14			
ATTENTION (A)	6957742.99	1	10.08	.007*	
A × G	56530.51	1	.08	.779	.06
Error	690499.01	14			
Interactions					
B × A	27816.59	1	.21	.652	.07
B × A × G	45835.97	1	.35	.564	.09
Error	131125.25	14			

Note. All controls were lifetime non-users of cannabis. Effect sizes are sreported in text and on figures. For abbreviations refer to Tables 1 and 2

*p < .05

**Fig. 2:**
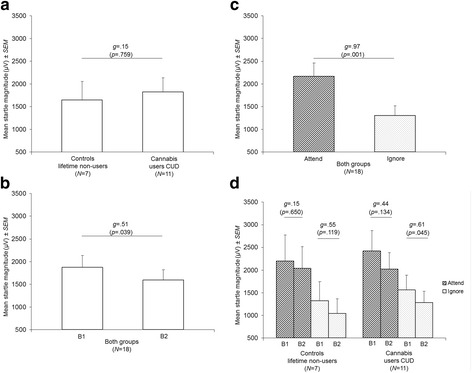
Mean startle magnitudes adjusted for nicotine and alcohol use and depending on **a** group (controls: lifetime non-users of cannabis vs. cannabis users with CUD), **b** block (1 vs. 2), **c** attention (Attend vs. Ignore Tasks), and **d** group, block, and attention. All *p*-values were adjusted using Bonferroni’s correction. Abbreviations: B1, Block 1; B2, Block 2; CUD, cannabis use disorder; *g*, standardized mean difference (Hedges’ *g*; effect size); *N*, sample size; *SEM*, standard error of the mean

The same pattern of responses as in the previous analysis was seen in controls (lifetime non-users of cannabis) and cannabis users with lifetime CUD. Specifically, startle magnitudes were similar in both groups (Fig. 2a). Startle habituation (with moderate effect size; Fig. 2b) and attentional modulation (with large effect size; Fig. 2c) occurred in both groups. Finally, startle habituation with moderate effect sizes was observed on the Ignore Task in both groups (Fig. 2d).

## Discussion

The current study shows that habituation of the acoustic startle reflex depends on selective attention but not on cannabis use or CUD in otherwise healthy samples from the general population. Although the statistical power to detect group differences was low based on small group sizes, the pattern and magnitude of startle responses were remarkably similar between healthy controls and cannabis users in our study (using selective attention paradigms) and also in other, uninstructed, studies [9, 19, 20]. It appears that startle habituation occurs in the absence of a specific attentional instruction in controls and cannabis users (some with CUD and in those at risk for psychosis) [9, 19, 20] or only when attention is directed away from pulses during a selective attention paradigm (current study). Therefore, unlike PPI, startle habituation may not be affected by any level of cannabis use. Specifically, startle habituation occurred in heavier users of cannabis using six days per week [20] and four days per week [19], following cannabis abstinence for 18 h to three days [19, 20], in users with urine positive for cannabinoids (our study, [9, 20]), and in users with lifetime CUD (our study and [20]). On the other hand, a high degree of heterogeneity in definition of cannabis use among all studies above might have masked any deficits in startle habituation. Future research with larger samples is necessary to assess startle habituation depending on the acute vs. long-term use and in reliably and validly classified low-level vs. heavy cannabis users.

Interestingly, startle habituation was present when participants ignored the auditory pulses and startle responses were similar in magnitude in controls, cannabis users, as well as in a pilot sample of non-users of cannabis with schizophrenia (data for latter not shown). Since the same pilot sample of schizophrenia patients and the same cannabis users showed a significant reduction in PPI when attending to pulses relative to controls [23], a trend towards an intact startle habituation in all three groups indicates that different neural processes might underlie startle habituation and PPI. In general, unlike the influence of attention on PPI, it seems that a minimal processing of pulses required while attention is being directed away from them, was necessary for startle habituation to occur in our study and also in another study using healthy participants [47].

The current results confirm that the startle reflex is modulated by selective attention in healthy controls and also in cannabis users. Both groups showed consistently reduced startle responses when attention was directed away from pulses compared to attending to pulses. A similar pattern of attentional modulation of startle magnitudes was shown in another study with healthy participants on the Visual Attention Task (equivalent to our Ignore Task) compared to the No Attention Task (passive attention equivalent to our Attend Task) and Auditory Attention Task (active attention) [47]. Intact attentional modulation of startle habituation in cannabis users is surprising considering that cannabis use affects selective attention [40]. Therefore, on the one hand, the attentional deficit associated with cannabis consumption might depend on a difficulty of an attentional task and be less severe than the attentional deficit observed in schizophrenia [23]. On the other hand, any impairments in selective attention might depend on factors, such as acute intoxication or the frequency/total duration of cannabis use [48] rather than on any level of cannabis use and/or presence of CUD (as in the current study). Therefore, future studies should investigate startle habituation during selective attention tasks taking into account task difficulty, acute intoxication, and heaviness of cannabis use.

There were a number of limitations in the current study. First, the statistical power to detect group differences was low due to only small sample sizes. Thus, the current results should be interpreted with caution and may not be generalisable to a wider population of cannabis users. Interestingly, the remarkably similar pattern of results (in terms of the effect sizes) suggests that at least a trend exists towards presence of startle habituation regardless of cannabis use. Second, as most other studies in this area, the current study was not specifically designed to focus on startle habituation. Our startle paradigm included pulse-alone and prepulse-and-pulse trials (excluded from the current analysis). Thus, startle habituation was inspected in blocks of pulse-alone trials rather than individual trials because of our complex study design (both attentional tasks started with different trials). We also included more trials per block than most other studies. An additional analysis using a three-block design produced similar results (not shown) to the results reported here. Thus, it is unlikely that a higher number of blocks with fewer trials would substantially alter our results. A study designed to best quantify startle habituation has shown that, similarly to trials included in our analysis, startle habituation occurred on trials 2–13 using 100 dB pulses [39]. Third, we have not controlled for the personality traits or the general cognitive performance. Although startle habituation was not related to the former in healthy controls [49], personality characteristics affected habituation rate in healthy participants [50]. Fourth, due to very low startle responses (mean peak magnitudes <10 μV) on all trials 23 % of all participants (*N* = 7 controls and *N* = 5 cannabis users) were classified as non-responders and were excluded from the study. The non-response rate probably resulted from deficiencies in detecting the EMG signal using skin-surface electrodes rather than from group membership because non-responders were found in both groups. Therefore, the electrode resistance should be measured prior to data collection to improve the quality of recording and reduce the non-response rate. Finally, although it cannot be ruled out, it is unlikely that acute cannabis use and presence of cannabinoids in urine affected the current results. There were no trends towards any associations between urine cannabinoids and startle habituation according to bivariate correlations (results not shown). However, future studies should investigate the impact of acute use of cannabis on startle habituation in larger samples.

## Conclusion

In summary, startle habituation appears to depend on selective attention but not on cannabis use or CUD. Startle habituation was present when attention was directed away from auditory startling pulses in healthy controls and cannabis users. Such similar pattern of results in both groups suggests that at least a trend exists towards presence of startle habituation regardless of cannabis use. The current results should be replicated in larger samples of cannabis users taking into account the effects of acute exposure as well as heaviness and duration of use. Since startle habituation occurs when attention is drawn away from rather than directed towards the startling stimuli, researchers should control for any differences in attention between groups when studying startle habituation.

## Abbreviations

CUD: Cannabis use disorder
PPI: Prepulse inhibition of the startle reflex
